# Spatiotemporal and Functional Characterisation of the *Plasmodium falciparum* cGMP-Dependent Protein Kinase

**DOI:** 10.1371/journal.pone.0048206

**Published:** 2012-11-05

**Authors:** Christine S. Hopp, Christian Flueck, Lev Solyakov, Andrew Tobin, David A. Baker

**Affiliations:** 1 Faculty of Infectious and Tropical Diseases, London School of Hygiene & Tropical Medicine, London, United Kingdom; 2 Department of Cell Physiology and Pharmacology, University of Leicester, Leicester, United Kingdom; Weill Cornell Medical College, United States of America

## Abstract

Signalling by 3′–5′-cyclic guanosine monophosphate (cGMP) exists in virtually all eukaryotes. In the apicomplexan parasite *Plasmodium*, the cGMP-dependent protein kinase (PKG) has previously been reported to play a critical role in four key stages of the life cycle. The *Plasmodium falciparum* isoform (PfPKG) is essential for the initiation of gametogenesis and for blood stage schizont rupture and work on the orthologue from the rodent malaria parasite *P. berghei* (PbPKG) has shown additional roles in ookinete differentiation and motility as well as liver stage schizont development. In the present study, PfPKG expression and subcellular location in asexual blood stages was investigated using transgenic epitope-tagged PfPKG-expressing *P. falciparum* parasites. In Western blotting experiments and immunofluorescence analysis (IFA), maximal PfPKG expression was detected at the late schizont stage. While IFA suggested a cytosolic location, a degree of overlap with markers of the endoplasmic reticulum (ER) was found and subcellular fractionation showed some association with the peripheral membrane fraction. This broad localisation is consistent with the notion that PfPKG, as with the mammalian orthologue, has numerous cellular substrates. This idea is further supported by the global protein phosphorylation pattern of schizonts which was substantially changed following PfPKG inhibition, suggesting a complex role for PfPKG during schizogony.

## Introduction

Malaria remains a major public health concern in Africa, Asia, and Latin America and the global number of deaths due to malaria was estimated to be 655,000 in 2010 [1], most of which are children under the age of five, although new data reports up to 1.24 million deaths caused by malaria in the same year [2]. Five unicellular parasites of the genus *Plasmodium* (*P. falciparum*, *P. vivax*, *P. ovale*, *P. malariae* and *P. knowlesi*) can infect humans and cause malaria, but *P. falciparum* is responsible for the most severe form of the disease. In the human host, following an initial phase of development in the liver, *P. falciparum* invades erythrocytes, where it matures from a ring to a trophozoite stage and nuclear division gives rise to the schizont stage, which releases merozoites ready to invade new red blood cells [3]. In mammalian cells, cGMP-signalling is involved in a broad range of cellular processes, such as calcium homeostasis, platelet activation, phototransduction and smooth muscle contraction [4]. In apicomplexan parasites, the components of the cGMP-pathway, comprising the cGMP-synthesising guanylyl cyclases and cGMP-hydrolysing phosphodiesterases, differ in their structural and regulatory properties from their mammalian homologues. In *Plasmodium*, PKG is believed to be the primary effector molecule of cGMP. Since *PKG* is refractory to gene disruption in *P. falciparum* (*PfPKG*; PlasmoDB identifier PF3D7_1436600) and *P. berghei*, an essential role for the enzyme in the asexual blood stages was assumed [5], [6]. We have previously used a chemical genetic approach to investigate PfPKG function [5], [7], [8]. The trisubstituted pyrrole 4-[2-(4-fluorophenyl)-5-(1-methylpiperidine-4-yl)-1*H*-pyrrol-3-yl] pyridine (compound 1) [9] and an even more potent inhibitor, the imidazopyridine compound 4-[7-[(dimethylamino) methyl]-2-(4-fluorophenyl) imidazo[1,2-*a*] pyridin-3-yl] pyrimidin-2-amine (compound 2) [10] selectively target PfPKG by virtue of their ability to utilise a unique active site-associated pocket, conferred by an unusually small gatekeeper residue. Use of these inhibitors in conjunction with a transgenic *P. falciparum* line, rendered inhibitor-resistant by substitution of the gatekeeper residue in the endogenous PfPKG enzyme with a more bulky one, allowed functional analysis of this kinase in previous studies. PfPKG was found to be essential in triggering gametogenesis [5] and work using *P. berghei* parasites has identified additional vital roles for PbPKG in ookinete differentiation and motility [6] and late liver stage development [11], [12]. In the asexual blood stages of *P. falciparum*, PKG inhibition was found to block schizont rupture [8]. Recently, PfPKG was reported to regulate the initiation of the proteolytic cascade prior to egress that involves the subtilisin-like serine protease 1 (PfSUB1) [13]. PfSUB1 is maximally expressed in late *P. falciparum* blood stage schizonts [14] and localises to dense granule-like structures, termed exonemes, that are present at the apical end of the individual merozoite [15]. Prior to egress, PfSUB1 is discharged into the parasitophorous vacuole, where it proteolytically cleaves the serine repeat antigens (PfSERAs) and components of the merozoite surface protein 1 (PfMSP1) complex [14], [16]. Processing of PfMSP1 was found to be blocked in schizonts treated with compound 1 [13].

Some uncertainty exists concerning the timing of expression and activity of PfPKG in the asexual blood stages of *P. falciparum*, likely due to methodological differences in the protocols used between studies [17]–[19]. It has been reported that PfPKG expression at the protein level is high in *P. falciparum* ring stage parasites, as well as in gametocytes, whereas in microarray data originating from *P. falciparum* strains in culture [20], as well as in the *P. falciparum* RNAseq data [21], *PfPKG* mRNA expression peaked in the late asexual blood stages of the parasite. Consistent with these latter data, maximal expression of PfPKG protein was found in the late trophozoite and schizont stage in a more recent study [8].

The apicomplexan parasites *Toxoplasma gondii* and *Eimeria tenella* have one *PKG* gene copy, but due to alternative translation start sites, each express two isoforms. While the shorter isoform is cytosolic, the full length protein, similar to the mammalian PKG-II, undergoes N-terminal myristoylation and palmitoylation, which mediates membrane anchoring [9]. The *Plasmodium* PKG is also encoded by a single gene, but there is no evidence of alternative start sites and it lacks the consensus amino acid motifs required for acylation [18]. In *P. berghei*, the endogenous copy of *PbPKG* has previously been tagged with green fluorescent protein and the fusion protein was found to localise to the cytosol [6]. When PfPKG was episomally expressed in *T. gondii* tachyzoites, the protein was also reported to have a cytosolic location [18]. Prior to this study, the cellular location of PfPKG in *P. falciparum* had not been examined.

Using epitope-tagged PfPKG in the present work, PfPKG expression was found to be maximal at the late schizont stage and appeared to have a primarily cytosolic location, but in late schizonts, a degree of colocalisation was detected with markers of the ER. In accordance with the microscopy results, PfPKG was largely soluble but partly associated with the peripheral membrane fraction in solubility assays. Treatment of schizonts with the specific PKG inhibitor compound 2 identified several PfPKG-dependent changes in the global protein phosphorylation pattern.

## Materials and Methods

### Transfection Plasmid Construction

Transfection constructs based on the pHH1 vector [22] contained a 1.7 kbp C-terminal fragment of the *PfPKG* gene and an in-frame C-terminal HA- [23] or PTP-tag [24], followed by a 0.65 kbp fragment of the 3′UTR of *PfPKG*. For detailed plasmid construction see supporting information (Methods S1). Following single crossover homologous recombination the PfPKG gene, under the control of the endogenous promoter, was tagged at the C-terminus and flanked by a segment of the endogenous 3′ untranslated region.

### *P. falciparum* Culture and Transfection

*P. falciparum* parasites of the clone 3D7 were cultivated in human A^+^ erythrocytes (National Blood Transfusion Service, UK) and RPMI 1640 medium (Invitrogen, Life Technologies) supplemented with 0.5% (w/v) albumax type II (Gibco, Invitrogen) according to standard procedures [25]. For transfection, ring-stage parasites were electroporated as described previously [26]. Following approximately four weeks of positive selection with 5 nM WR99210 (a kind gift from Jacobus Pharmaceuticals, New Jersey, USA), transformants were subjected to three cycles of three weeks on/off drug to encourage loss of episomal transformants and selection of parasites in which integration had taken place. Parasites were cloned by limiting dilution and clonal cultures were genotyped by PCR and Southern blotting according to standard procedures [27]. For details on primers and probes see supporting information (Methods S1).

### Preparation of Parasite Lysates and Subcellular Fractions

For analysis of the temporal PfPKG expression profile, parasites were released with 0.15% saponin and lysed in HBS buffer (50 mM HEPES pH 7.4, 1 mM EDTA, 1% Nonidet P-40, 10 mM NaF, 0.1 mM sodium orthovanadate, 1 mM DTT) supplemented with 1 mM PMSF, 10 µg/ml leupeptin, 10 µg/ml antipain and 10 µg/ml pepstatin A and samples were subjected to SDS-PAGE and Western blotting according to standard procedures. The protocol for sequential solubilisation of parasite proteins was adapted from [28]. Briefly, saponin-released late *P. falciparum* blood stages were resuspended in hypotonic lysis buffer (5 mM Tris-HCl pH 8.0), freeze-thawed and the supernatant containing the soluble protein fraction was collected after centrifugation at 16000 g for 15 minutes at 4°C. The pellet was washed once and resuspended in 0.5 pellet volume of DNAse digestion buffer (5 mM Tris-HCl, 10 mM MgCl_2_, supplemented with DNAse I) and incubated for 10 minutes at room temperature. 0.1 M Na_2_CO_3,_ pH 11.0 was added and the suspension incubated for 30 minutes on ice and centrifuged as above. The supernatant was saved as the peripheral membrane protein fraction. The remaining pellet was washed and extracted with 4% SDS/0.5% TritonX-114 in 0.5×PBS to solubilise integral membrane proteins. Buffers were supplemented with complete EDTA-free protease inhibitor cocktail (Roche). All extractions were performed in 5 saponin pellet volumes and equal volumes of the three supernatants were analysed by SDS-PAGE and Western blotting according to standard procedures.

### Immunofluorescent Analysis (IFA)

Standard thin blood films were fixed in 4% formaldehyde, permeabilised in 0.1% TritonX-100 and incubated with antibodies diluted in 1×PBS containing 3% BSA. Slides were mounted in Hydromount with DAPI (National Diagnostics), before pictures were aquired on a LSM 510 Axiovert confocal microscope (Zeiss) and analysed with LSM Image Browser software (Zeiss).

### Antibodies

The rabbit anti-human PKG (PK1018, Calbiochem) and rat anti-HA (clone 3F10, Roche) antibodies were diluted 1∶5000 for Western blotting and 1∶50 for IFA. The rabbit anti-ProtC (ab18591-200, Abcam) was used at 1∶400 in Western blots. Mouse monoclonal anti-Pfαtubulin (Tat1) (provided by Keith Gull, University of Oxford) was used in Western blots at 1∶20000. The mouse anti-PfPMV (obtained from the Malaria Research and Reference Reagent Resource Center, MR4) was used at 1∶2500 in Western blots and at 1∶20 in IFA. The mouse anti-PfGAPDH (obtained from Claudia Daubenberger, Swiss Tropical and Public Health Institute) was diluted 1∶20000 for Western blots and 1∶1000 for IFA. In IFA, the following antibody dilutions were being used: mouse anti-HA (clone 16B12, Convance) at 1∶2000, rabbit anti-PfGAP45 and rat anti-PfBiP (obtained from Anthony A. Holder, NIMR) at 1∶1000, rabbit anti-PfRab11A (provided by Gordon Langley, Inserm U1016) at 1∶200 and rabbit antibodies against PfAMA1, PfSUB1 (obtained from Michael Blackman, NIMR) at 1∶200. For Western blots, secondary antibodies were HRP-coupled goat anti-rabbit (1∶6000) (Dako), goat anti-mouse (1∶2500) (Dako) and goat anti-rat-HRP (1∶1000) (SC-2006, Santa Cruz). For IFA the following secondary antibodies were used at a dilution of 1∶200: Alexa Fluor 594 donkey anti-rabbit IgG with minimal cross-reactivity (A21207, Invitrogen), Alexa Fluor 488 goat anti-rat IgG, pre-absorbed against mouse IgG (A11006, Invitrogen), Alexa Fluor 488 donkey anti-mouse IgG, highly cross-absorbed (A21202, Invitrogen) and Alexa Fluor 594 goat anti-mouse IgG, highly cross-adsorbed (A11032, Invitrogen).

### Metabolic ^32^P-orthophosphate Labelling of *P. falciparum* Schizonts

Synchronised *P. falciparum* schizonts were purified by magnetic activated cell sorting (MACS) and eluted in 1×Krebs buffer (11.8 mM NaCl, 4.7 mM KCl, 1.2 mM KH_2_PO_4_, 1.2 mM MgSO_4_, 4.2 mM NaHCO_3_, 10 mM glucose, 2 mM CaCl_2_, 10 mM HEPES pH 7.4). Parasites were pre-treated with 4 µM compound 2 (0.1% final DMSO) or 0.1% DMSO only, for 1 hour and incubated for an additional hour in 1×Krebs containing 50 µCi/ml ^32^P-orthophosphate (Perkin Elmer LAS). Schizonts were then harvested by centrifugation and lysed in 1% NP-40 lysis buffer. Protein samples were fractionated using anion-exchange chromatography (ÄKTA system, GE Healthcare) and fractions 10–14 were analysed by SDS-PAGE/autoradiography.

## Results

### PfPKG Expression Peaks in the Late Asexual Blood Stages of *P. falciparum*

**Figure 1:**
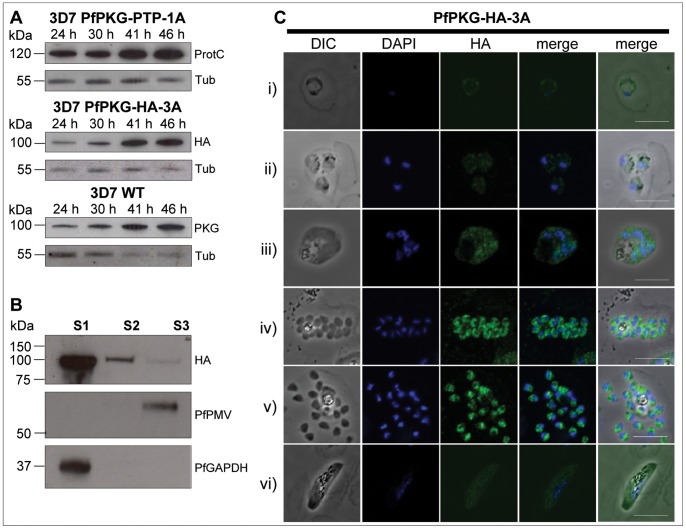
PfPKG expression peaks in late blood stages and is carbonate-soluble. (**A**) Western blots of synchronised cultures of the PfPKG-HA-3A clone and WT parasites (3D7 clone), 24 hours (mostly mid trophozoites), 30 hours (mostly late trophozoites), 41 hours (mostly early schizonts) and 46 hours (mostly late schizonts) post invasion were detected with anti-HA and anti-humanPKG, respectively. Blots were re-probed with an antibody against Pfαtubulin to estimate the relative total protein loading between lanes. (**B**) Sequential solubilisation of parasite proteins from saponin-released late trophozoites and schizonts. S1: soluble protein fraction (5 mM Tris-HCl, freeze thaw); S2: peripheral membrane fraction (extraction with 100 mM Na_2_CO_3_); S3: integral membrane fraction (extraction with 4% SDS/0.5% TX-114/0.5×PBS). Equal volumes of the three supernatants were analysed by SDS-PAGE and Western blots were probed for the integral membrane protein PfPMV [31], stripped and re-probed simultaneously for PfGAPDH [30] and PfPKG-HA. Densitometric analysis of the scan of the blot presented revealed that 89.3% of PfPKG-HA is present in fraction S1, while fractions S2 and S3 contain 9.5% and 1.2%, respectively. (**C**) Immunofluorescent anti-HA detection in fixed smears of erythrocytic stages of the PfPKG-HA-3A clone. Representative images of (i) a ring stage parasite, (ii) three early trophozoites, (iii) an early schizont, (iv, v) late schizonts (approximate hours post invasion: (i) 4–10, (ii) 20–26, (iii) 33–39, (iv, v) 45–48) and (vi) a stage III gametocyte are shown together with bright field images (first column) and parasite nuclei stained with DAPI (second column). Bars ∼5 µM.

*P. falciparum* parasites expressing triple HA- (haemagglutinin) [23], as well as PTP- (protein C - tobacco etch virus - protein A) [24] C-terminally-tagged PfPKG from the endogenous locus were produced via allelic replacement. Southern blot analysis of clones PfPKG-HA-3A and PfPKG-PTP-1A confirmed that the plasmid had integrated through the expected single-crossover homologous recombination event, since bands of the predicted sizes were detected (Fig. S1). Expression of epitope-tagged PfPKG was verified by Western blotting and epitope-tagged proteins of the expected sizes were detected, as well as two bands of lower molecular weight, most likely corresponding to PfPKG degradation products (Fig. S2). The temporal expression profile of PfPKG-HA fusion protein was investigated throughout the asexual blood stage by Western blotting using synchronised cultures. Samples were taken at 24 hours (mostly mid trophozoites), 30 hours (mostly late trophozoites), 41 hours (mostly early schizonts) and 46 hours (mostly late schizonts) post invasion. Relative to Pfαtubulin, PfPKG expression increased as the parasites matured and peaked in late schizont stages (Fig. 1A). A similiar expression pattern was observed for the PfPKG-PTP fusion protein (Fig. S2).

Notably, the expression profile of epitope-tagged fusion proteins correlated with that of wild type (WT) PfPKG detected with a commercial PKG antibody, raised against a synthetic peptide corresponding to the C-terminal peptide of human PKG. This antibody detects a protein of approximately 98 kDa, corresponding to WT PfPKG, as reported previously [5], [18]. While the PfPKG-PTP fusion protein was still detectable by this peptide antibody, HA-tagging of PfPKG ablated reactivity with the antibody (Fig. S2), possibly as a result of a masking of the epitope by the proximal C-terminal HA-tag. This provides convincing evidence that this mammalian PKG antibody is reacting specifically with PfPKG in the parasite.

### PfPKG is Largely Soluble, but Shows Some Association with the Peripheral Membrane Fraction

Assessing the subcellular location of a protein kinase is an important preliminary step to determine its function and is relevant to the identification of potential substrates. To investigate whether PfPKG is cytosolic or bound to membranes, sequential extraction of parasite proteins in hypotonic, carbonate and SDS/TX-114 buffers was performed. While hypotonic lysis solubilises cytosolic proteins, carbonate treatment releases proteins that are indirectly attached to membranes by e.g. binding to integral membrane proteins, whereas integral membrane proteins are carbonate-insoluble and remain in the membrane fraction [29]. Most of the HA-tagged PfPKG was solubilised during hypotonic lysis (Fig. 1B), arguing for a mainly cytosolic location of the protein. The subsequent carbonate treatment, which solubilises peripheral membrane proteins, released virtually all of the remaining PfPKG protein. Glyceraldehyde-3-phosphate dehydrogenase (PfGAPDH) used as a cytosolic marker [30], was entirely solubilised by the hypotonic lysis. Absence of PfGAPDH from the carbonate fraction suggests that the PfPKG protein found in this fraction reflects a true membrane association rather than carry over or incomplete hypotonic lysis. An integral membrane protein, the protease plasmepsin V (PfPMV) [31], which is located in the ER-membrane, was resistant to carbonate extraction, as demonstrated previously [31] and was solubilised upon SDS/TX-114-extraction of the membranes after carbonate treatment.

### PfPKG Location Overlaps with that of Cytoplasmic and ER Protein Markers

**Figure 2:**
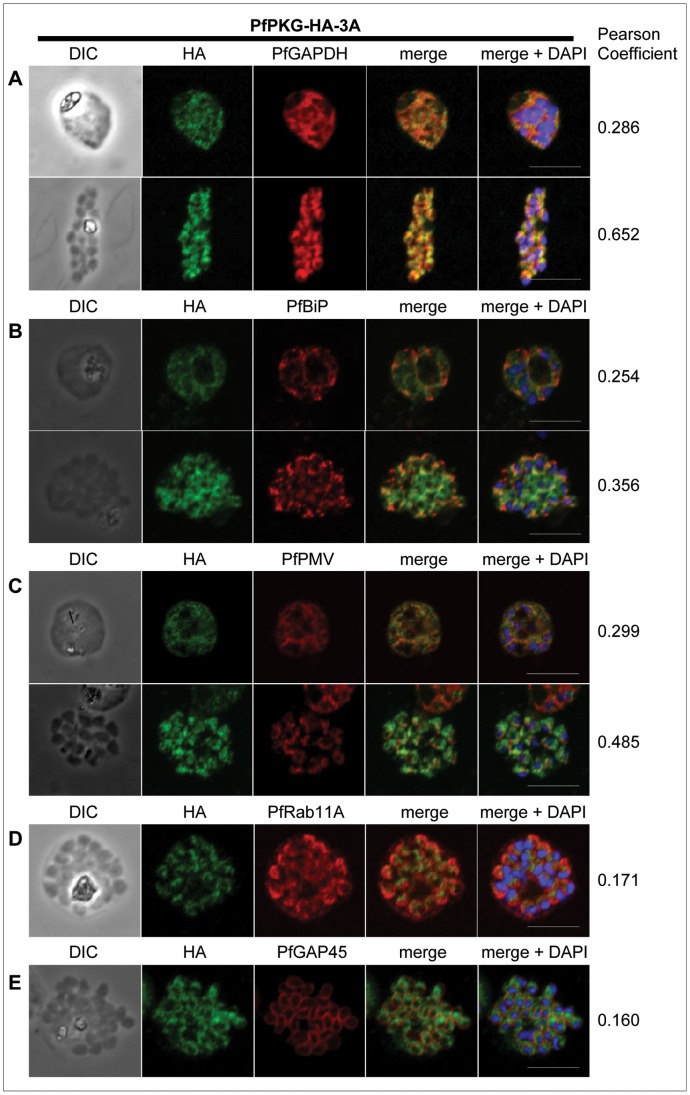
Subcellular location of PfPKG in mature schizonts. Dual immunofluorescent detection of PfPKG-HA in fixed smears of early and late schizonts of the PfPKG-HA-3A clone together with (**A**) PfGAPDH [30], (**B**) PfBiP [32], (**C**) PfPMV [31], (**D**) PfRab11A [33] and (**E**) PfGAP45 [34]. Representative images are shown for each antibody, together with bright field images (first column) and parasite nuclei stained with DAPI (in the merged image). Bars ∼5 µM. To quantify co-localisation, Pearson coefficients [36] of the individual stains were calculated using Imaris image analysis software (Bitplane).

The subcellular location of PfPKG was investigated by IFA using the parasite clone PfPKG-HA-3A. Consistent with Western blotting results, epitope-tagged PfPKG was detected most strongly in late blood stage parasites (Fig. 1C). A diffuse pattern of relatively low level PfPKG expression was detected in ring and early trophozoite stages and gametocytes (Fig. 1C). Very similar results were obtained using PfPKG-PTP-1A clones, as well as the parental 3D7 parasites in conjunction with the commercial human PKG-I antibody (Fig. S3). The HA/ProtC antibodies did not react with WT 3D7 *P. falciparum* in IFA (Fig. S4). In very mature, segmented schizonts, PfPKG appeared to be residing in the cytoplasm of the individual merozoite and the staining was maximal in the perinuclear area (Fig. 1C). To evaluate the subcellular location of PfPKG in blood-stage parasites, dual labelling of HA-tagged PfPKG in the PfPKG-HA-3A clone, together with protein markers of known subcellular compartments was performed. Significant overlap between the PfPKG-HA staining and the pattern obtained with an antibody detecting PfGAPDH [30] in early and late blood stage schizonts (Fig. 2A) suggests that like PfGAPDH, PfPKG is primarily found in the cytosol. However, the PfPKG-HA staining also partially overlapped with that of the ER membrane protein PfPMV [31] and that of the ER lumen marker PfBiP (binding immunoglobulin protein) [32], (Fig. 2B and C) in both early and late schizonts. Downstream of the ER in the secretory pathway, the small G-protein PfRab11A is thought to be involved in vesicle trafficking and localises to the rhoptries and the inner membrane complex (IMC) at the apical end of the merozoite [33]. In mature schizonts, the location of PfPKG-HA appeared to be largely distinct from that of PfRab11A (Fig. 2D). Furthermore, PfPKG-HA appeared to be absent from the IMC in segmented schizonts, since no co-localisation of the IMC marker, the glideosome associated protein 45 (PfGAP45) [34] and PfPKG-HA was observed (Fig. 2E). To investigate PfPKG-HA location in relation to the Golgi apparatus, a dual staining of PfPKG-HA and PfErd2 [35], was performed and distinct staining patterns were obtained, with PfErd2 confined to a precise punctate location reported as a single Golgi cisterna close to the nucleus of the parasite [35] (Fig. S6).

Calculation of the Pearson coefficient [36] is used for the quantitative evaluation of the degree of overlap between two staining patterns. Using the Imaris image analysis software (Bitplane) co-localisation of PfPKG-HA with the various marker proteins was quantified as 0.286/0.652 for PfGAPDH (trophozoite/schizont), 0.299/0.485 for PfPMV (trophozoite/schizont), 0.254/0.356 for PfBiP (trophozoite/schizont), 0.171 for PfRab11A (schizont) and 0.160 for PfGAP45 (schizont) (mean Pearson coefficients, n = 2 to 4 parasites, calculated with a 25% threshold). In conclusion, analysis of these dual staining experiments suggests a primarily cytosolic localisation of PfPKG, coinciding partially with the ER.

Immunoelectron microscopy is a powerful technique used to reveal the cellular location of a protein and was attempted with the PfPKG-HA-3A clone in conjunction with an anti-HA antibody; however the staining pattern was not conclusive. Gold particles were visualised in the cytosol of the merozoite, but also adjacent to membranes (arrowed in Fig S5). No signal was obtained in the secondary-only control sample (data not shown).

### Normal Localisation of PfGAP45, PfSUB1 and PfAMA1 in PKG Inhibitor-treated *P. falciparum* Blood Stage Schizonts

**Figure 3:**
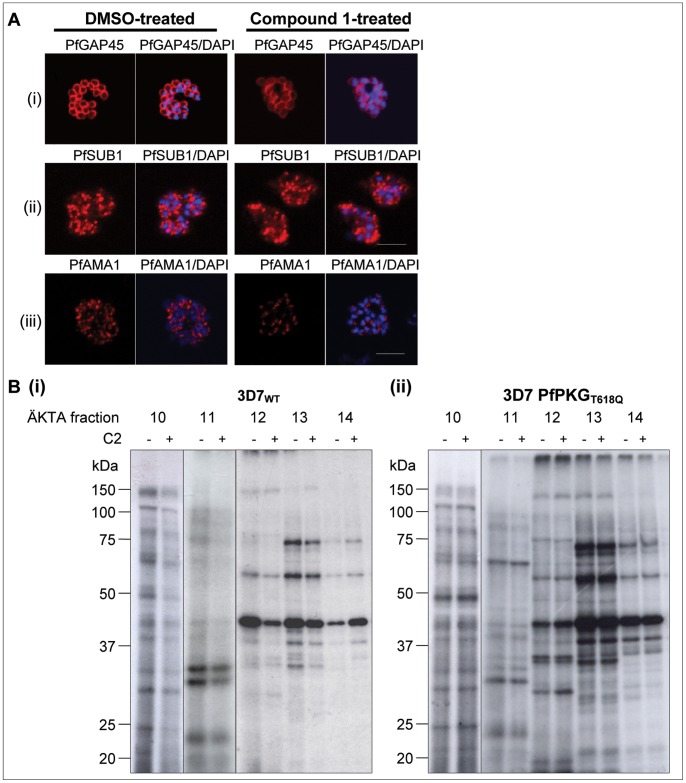
Parasite morphology and global protein phosphorylation pattern of PKG inhibitor-treated *P. falciparum* schizonts. (**A**) Immunofluorescent staining of DMSO/compound 1-treated WT schizonts using antibodies detecting (i) PfGAP45 [34], (ii) PfSUB1 [14] and (iii) PfAMA1 [23]. Representative images are shown for each staining together with parasite nuclei stained with DAPI. Bars ∼5 µM. (**B**) Metabolic labelling of phosphoproteins in *P. falciparum* schizonts. Autoradiographs of (i) 3D7 WT and (ii) gatekeeper mutant 3D7 PfPKG_T618Q_ schizonts, treated with ^32^P-orthophosphate and DMSO (−) or compound 2 (+) prior to lysis, ÄKTA anion exchange chromatography (fractions 10–14 are shown) and separation by SDS-PAGE. Rectangular boxes highlight bands that show a differential signal following inhibitor-treatment in WT, but not PfPKG_T618Q_ schizonts.

Recent data showed that *P. falciparum* blood stage parasites that are treated with the PKG inhibitor compound 1 develop normally up to the schizont stage, but they are unable to rupture [8]. In the same study, the morphology of Giemsa-stained mature schizonts after prolonged (24 h) treatment with 2 µM compound 1 was found to be abnormal, with stronger separation of the individual merozoites compared to untreated schizonts [8]. However, localisation of PfMSP1 and the rhoptry neck protein 4 (PfRON4, Pf225) in compound 1-treated parasites was found to be normal in IFA [8]. In the present study, the location of three schizont proteins was assessed using IFA, to further evaluate the integrity of subcellular compartments of the blood stage schizont and trafficking of these proteins following treatment with the PKG inhibitor compound 1. Antibodies against the IMC marker PfGAP45 [34], the exoneme residing PfSUB1 [14], and the microneme marker apical membrane antigen 1 (PfAMA1) were used. Segmented *P. falciparum* schizonts were treated for 6 hours with 4 µM compound 1 prior to immunostaining. Overall, there were no marked morphological differences (assessed by Giemsa staining, data not shown) between treated and untreated schizonts and all marker proteins appeared to localise normally following compound 1-treatment (Fig. 3A), suggesting that PfPKG function is not involved in the control of the cellular trafficking to their respective compartments.

### PfPKG-dependent Changes in the Global Protein Phosphorylation Pattern in *P. falciparum* Blood Stage Schizonts

While PfPKG inhibitor-treated schizonts appear morphologically normal, merozoite egress is blocked. To investigate the extent of PfPKG biochemical activity at this life cycle stage, PfPKG-dependent changes in the global protein phosphorylation profile were revealed by analysis of metabolically ^32^P-orthophosphate-labelled *P. falciparum* segmented asexual blood stage schizonts, treated with 4 µM compound 2. Parasite lysates were fractionated via ion-exchange chromatography and separated by SDS-PAGE. Changes in the phosphorylation status of up to 20 protein bands were seen following treatment with compound 2 in WT 3D7 parasites. These changes were not observed in compound 2-insensitive parasites with the gatekeeper mutation (PfPKG_T618Q_), a result that firstly confirms the selectivity of compound 2 for PfPKG, but also points to a complex pattern of PfPKG-dependent phosphorylation events. Importantly, in addition to decreases in phosphorylation events following PfPKG inhibition with compound 2, there were also increases in phosphorylation. This complex pattern of phosphorylation changes following inhibition of PfPKG indicates that this kinase is likely to be in a key position in protein phosphorylation cascades. As reported previously, PfPKG is known to undergo autophosphorylation [17], [37]. However, no clearly labelled protein band at the size of PfPKG (approximately 98 kDa [5], [18]) was observed which is most likely due to the fact that only the most abundant phosphoproteins will be detected by autoradiography. In fraction 12, a prominent phosphoprotein of approximately ∼40 kDa was observed. While this band may represent parasite proteins previously identified as phosphorylated in global phospho-proteomic studies [38], [39] such as PfGAP45, 60S ribosome subunit and the 26S proteasome regulatory subunit, the precise identity can not be determined. We are currently attempting to identify PfPKG substrates using mass spectrometry approaches.

## Discussion

Since an important role for PfPKG in egress of merozoites from blood stage schizonts had previously been described [8], [13], this project aimed at characterising PfPKG further by assessing the temporal expression profile of the enzyme and its subcellular localisation, to identify precisely when and where in the parasite PfPKG is performing its role. Also, schizont morphology and phosphorylation events in response to treatment with specific PKG inhibitors were examined. In transgenic *P. falciparum* parasites, HA- and PTP-tagged PfPKG expression was detectable throughout the erythrocytic stages of the life cycle, but was found to be maximal at the segmented schizont stage in both Western blot and IFA. These data are in agreement with maximal *PKG* mRNA expression measured in mature blood stage schizonts of *Plasmodium* parasites according to available transcriptome data [20], [21], [40] and are consistent with the role of PfPKG in schizont rupture [8].

Subcellular fractionation and IFA analysis suggested that PfPKG has a primarily cytosolic location. In segmented schizonts, PfPKG staining was maximal in the area surrounding the nuclei and in dual staining experiments, there was marked overlap of PfPKG-HA with the ER-marker proteins PfBiP and PfPMV. This was reflected in a higher Pearson coeffient for these co-staining experiments, than with, for example, PfRab11A, a marker of vesicle trafficking [33].

The absence of the N-terminal acylation motifs in PfPKG, which are present in *Toxoplasma* and *Eimeria* PKG orthologues, strongly suggests that PfPKG is unlikely to be directly anchored to parasite membranes. In the present study, solubilisation experiments support this notion since carbonate extraction solubilised HA-tagged PfPKG. While the integral membrane protein PfPMV was found in the SDS/TX-114-fraction of membranes following carbonate treatment, only traces of PfPKG were found in this fraction, suggesting that the kinase does not behave as an integral membrane protein. However, the presence of PfPKG-HA in the peripheral membrane fraction after hypotonic lysis suggests that the kinase may be partially associated with the membrane in late schizonts through protein-protein interaction with a membrane-bound protein or complex. Together with the IFA findings, this suggests a potential location of PfPKG in the proximity of the early secretory pathway in late stage *P. falciparum* schizonts and possibly an interaction with ER membranes through an ER-localised protein complex.

The potential role of PfPKG in regulating assembly of specific cellular compartments in schizonts was analysed microscopically following treatment of parasites with compound 1. Although compound 1 inhibits *P. falciparum* merozoite egress, the integrity of IMC, exonemes and micronemes appeared to be unaffected, suggesting that the block likely occurs after differentiation of these cellular compartments. This is interesting, since it was recently found that PfPKG function is required for successful processing of PfMSP1 by PfSUB1 [13]. In conjunction with the present results, it is tempting to speculate that PfPKG regulates the activation or release of PfSUB1, rather than its cellular trafficking to the exonemes. The previously reported effect of greater separation of *P. falciparum* merozoites in schizonts after compound treatment [8] was not observed in the present study, where treatment time was 6 hours rather than 24 hours [8]. This longer treatment exposed schizonts that are mature, but unable to egress, to the drug in culture for several hours, most likely accounting for the stronger morphological differences between treated and untreated schizonts.

Analysis of the global phospho-proteome using fractionation of metabolically labelled parasites treated with compound 2 indicated that inhibition of PfPKG resulted in changes in the phosphorylation status of over 20 parasite proteins. The changes observed in the phosphoproteome following PfPKG inhibition were seen to be complex, with both increases as well as decreases in phosphorylation. Notably very few changes were seen in parasites expressing the inhibitor insensitve PfPKG_T618Q_ mutant, indicating that compound 2 had few off-target effects. The expression and sub-cellular localisation data reported here are consistent with a role for PfPKG in egress and the extensive impact of inhibition of PfPKG on the phospho-proteome indicates that this kinase likely has a complex interaction network to effect its role in schizont rupture and merozoite egress.

## Supporting Information

Figure S1 **Endogenous tagging of the** ***PfPKG*** **locus by allelic replacement. (A) Allelic replacement strategy.** Schematic representations of transfection plasmids and the *PfPKG* loci before and after individual integration of the tagging constructs (i, ii) are shown. The C-terminal 1.7 kbp of the *PfPKG* gene (grey shaded area) facilitated integration by single crossover homologuous recombination and was followed by the PTP−/HA-tag (striped/dotted area) and the 0.65 kbp fragment of the PfPKG 3′UTR. Arrowheads indicate primer binding sites. *Eco*RI and *Xba*I restriction sites (E and Xb), as well as hybridisation sites of Southern blotting probes to *PfPKG* (line) and *hDHFR* (dashed line) are shown. Integration of two plasmid copies into the *PfPKG* locus is depicted. **(B) PCR analysis of transfected cultures confirming integration.** The 5′ crossover junction was analysed by PCR using a 5′ primer hybridising to the PTP- and HA-tag, respectively and a 3′ primer bound the *PfPKG* locus upstream of the 1.7 kbp fragment that is present in the plasmid (for primer binding sites see arrowheads in (A)). Products can only be amplified in case of integration of the construct. Parental WT 3D7 parasite gDNA was used as a negative control for each primer set (PTP, HA). Sizes of obtained PCR products were as expected: 2.3 kbp (PfPKG-PTP), 1.9 kbp (PfPKG-HA). **(C) Southern blot analysis of gDNA from cloned parasite lines.** gDNA was digested with *Eco*RI and *Xba*I respectively. The *PfPKG* fragments detected were as expected for parental WT 3D7 (12.3 kbp) and PfPKG-PTP-1A (9.6 kb, 4.4 kbp and 6.3 kbp) and PfPKG-HA-3A (12.7 kbp, 7.8 kbp and 6.3 kbp) parasites. Presence of bands of the plasmid size (4.4 kbp in the PfPKG-PTP-1A parasites and 7.8 kbp in the PfPKG-HA-3A parasites) indicated that integration of more than one plasmid copy had occurred and elevated intensity of those plasmid bands suggested integration of multiple copies, a phenomenon which has previously been documented [14] and is not surprising, since plasmid copy numbers exceed the number of genomes during recombination [26]. Fragments of the *hDHFR* locus were 0.8 kbp and 1.7 kbp, as expected. Growth rate compared to the parental 3D7 line was not measured, but cloned lines displayed no evident growth defect upon daily culturing.
(TIF)

Figure S2 **Transgenic** ***P. falciparum*** **lines express tagged PfPKG.** Western blots of late blood stages of PfPKG-PTP-1A and PfPKG-HA-3A parasite clones and parental WT parasites of the 3D7 clone were probed with antibodies against **(A)** ProtC, **(B)** HA and **(C)** humanPKG. The epitope-tagged forms of PfPKG-PTP and PfPKG-HA, as well as two bands of lower molecular weight, most likely corresponding to PfPKG degradation products, are only detected in the corresponding clones, but not in the WT parasites. The human PKG antibody detects the WT PfPKG and the PfPKG-PTP fusion protein, but does not react with the epitope-tagged PfPKG-HA. The size-shift of tagged PfPKG species compared to the parental WT PfPKG (97.7 kDa) is consistent with the 18.7 kDa mass of the PTP-tag and 3.3 kDa for the HA-tag, which results in a total size of 116.4 kDa for PfPKG-PTP and 100.9 kDa for the PfPKG-HA fusion protein. As a protein loading control, Pfαtubulin was detected using a mouse monoclonal antibody (Tat1). **(D)** Western blot of synchronised parasite cultures of the clone PfPKG-PTP-1A 24 hours (mostly mid trophozoites), 30 hours (mostly late trophozoites), 41 hours (mostly early schizonts) and 46 hours (mostly late schizonts) post invasion was detected with anti-ProtC and re-probed with an antibody against Pfαtubulin to estimate the relative total protein loading between lanes.
(TIFF)

Figure S3 **IFA of PfPKG in PfPKG-PTP-1A and WT 3D7 lines.** Immunofluorescent detection using antibodies against **(A)** human PKG on WT parasites of the 3D7 clone and **(B)** ProtC on parasites of the PfPKG-PTP-1A clone. Representative images of (i) a ring stage parasite, (ii) two early trophozoites, (iii) an early schizont, (iv, v) late schizonts and (vi) a stage III gametocyte are shown together with bright field images (first column) and parasite nuclei stained with DAPI (second column). Bars ∼5 µM.
(TIF)

Figure S4 **Negative control IFA.** Immunofluorescent staining of WT parasites (clone 3D7) with **(A)** the rat HA-antibody, **(B)** the mouse HA-antibody and **(C)** the rabbit ProtC-antibody. Representative IFA images are shown together with bright field images (first column) and parasite nuclei stained with DAPI (second column). Bars ∼5 µM.
(TIF)

Figure S5 **Dual staining of PfPKG-HA together with PfERD2.** Dual staining of PfPKG-HA in fixed smears of early and late schizonts of the PfPKG-HA-3A clone together with PfErd2 [35]. Representative images are shown, together with bright field images (first column) and parasite nuclei stained with DAPI (in the merged image). Bars ∼5 µM.
(TIF)

Figure S6 **Immunoelectron microscopic visualisation of PfPKG-HA-3A.** Segmented schizonts of the PfPKG-HA-3A clone were fixed in formaldehyde/glutaraldehyde and mounted in LR white resin. PfPKG-HA was detected with mouse HA antibody; secondary detection was performed with 10 nM gold particle-coupled anti-mouse antibody. Sections were counter-stained with uranyl acetate and images were captured digitally on a Jeol JEM –1200EX II electron microscope. Bars 500 nm.
(TIF)

Methods S1 Additional details of experimental procedures.
(DOCX)
