# Supplementary figures and images for: Spatiotemporal and Functional Characterisation of the Plasmodium falciparum cGMP-Dependent Protein Kinase
Source: PLoS One. 2012 Nov 5;7(11):e48206. doi: 10.1371/journal.pone.0048206 (PMC3489689; doi:10.1371/journal.pone.0048206)

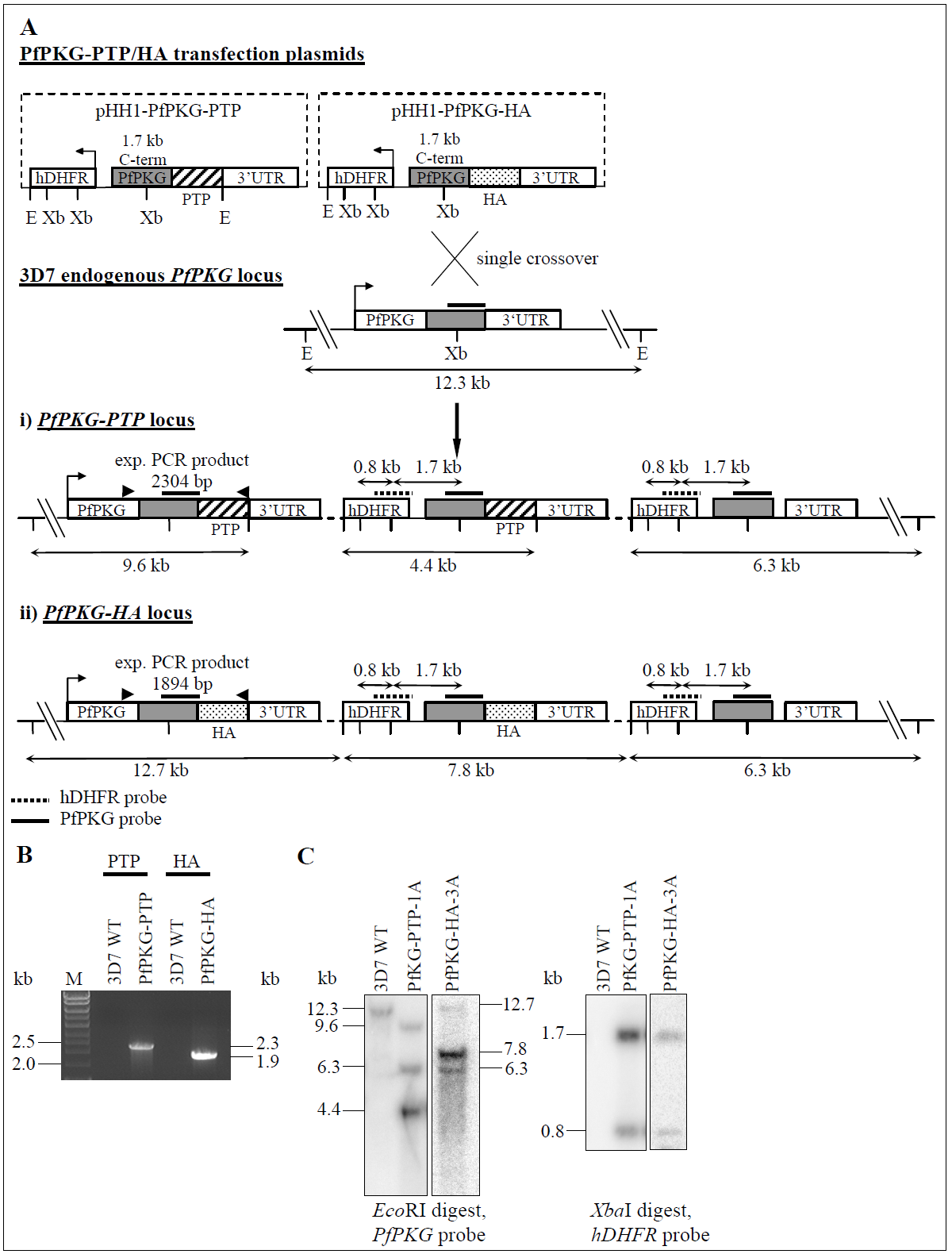

Supplement: Figure S1 — Endogenous tagging of the PfPKG locus by allelic replacement. (A) Allelic replacement strategy. Schematic representations of transfection plasmids and the PfPKG loci before and after individual integration of the tagging constructs (i, ii) are shown. The C-terminal 1.7 kbp of the PfPKG gene (grey shaded area) facilitated integration by single crossover homologuous recombination and was followed by the PTP−/HA-tag (striped/dotted area) and the 0.65 kbp fragment of the PfPKG 3′UTR. Arrowheads indicate primer binding sites. EcoRI and XbaI restriction sites (E and Xb), as well as hybridisation sites of Southern blotting probes to PfPKG (line) and hDHFR (dashed line) are shown. Integration of two plasmid copies into the PfPKG locus is depicted. (B) PCR analysis of transfected cultures confirming integration. The 5′ crossover junction was analysed by PCR using a 5′ primer hybridising to the PTP- and HA-tag, respectively and a 3′ primer bound the PfPKG locus upstream of the 1.7 kbp fragment that is present in the plasmid (for primer binding sites see arrowheads in (A)). Products can only be amplified in case of integration of the construct. Parental WT 3D7 parasite gDNA was used as a negative control for each primer set (PTP, HA). Sizes of obtained PCR products were as expected: 2.3 kbp (PfPKG-PTP), 1.9 kbp (PfPKG-HA). (C) Southern blot analysis of gDNA from cloned parasite lines. gDNA was digested with EcoRI and XbaI respectively. The PfPKG fragments detected were as expected for parental WT 3D7 (12.3 kbp) and PfPKG-PTP-1A (9.6 kb, 4.4 kbp and 6.3 kbp) and PfPKG-HA-3A (12.7 kbp, 7.8 kbp and 6.3 kbp) parasites. Presence of bands of the plasmid size (4.4 kbp in the PfPKG-PTP-1A parasites and 7.8 kbp in the PfPKG-HA-3A parasites) indicated that integration of more than one plasmid copy had occurred and elevated intensity of those plasmid bands suggested integration of multiple copies, a phenomenon which has previously been documented [14] and is not surprising, since plasmid [file pone.0048206.s001.tif]

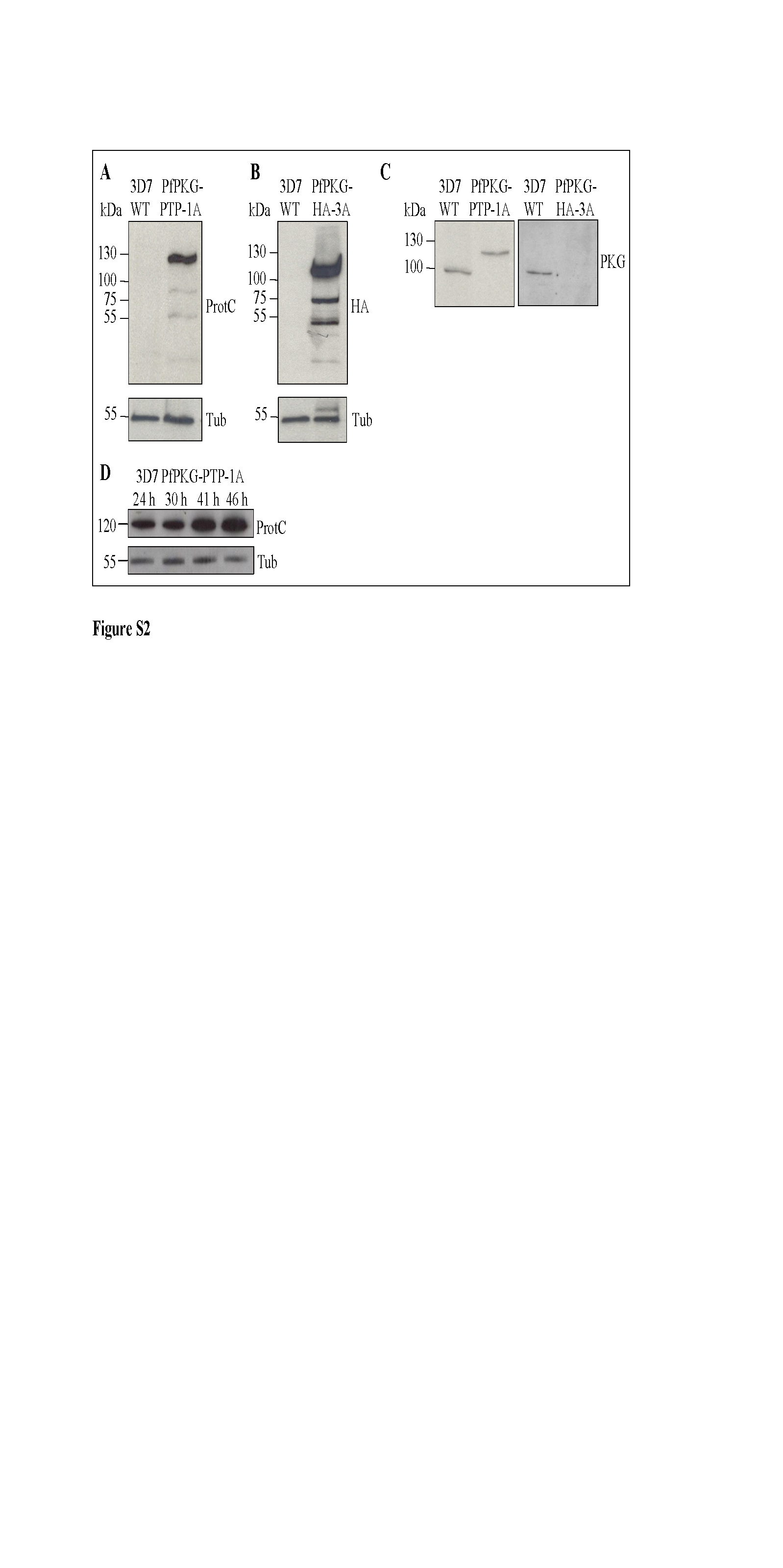

Supplement: Figure S2 — Transgenic P. falciparum lines express tagged PfPKG. Western blots of late blood stages of PfPKG-PTP-1A and PfPKG-HA-3A parasite clones and parental WT parasites of the 3D7 clone were probed with antibodies against (A) ProtC, (B) HA and (C) humanPKG. The epitope-tagged forms of PfPKG-PTP and PfPKG-HA, as well as two bands of lower molecular weight, most likely corresponding to PfPKG degradation products, are only detected in the corresponding clones, but not in the WT parasites. The human PKG antibody detects the WT PfPKG and the PfPKG-PTP fusion protein, but does not react with the epitope-tagged PfPKG-HA. The size-shift of tagged PfPKG species compared to the parental WT PfPKG (97.7 kDa) is consistent with the 18.7 kDa mass of the PTP-tag and 3.3 kDa for the HA-tag, which results in a total size of 116.4 kDa for PfPKG-PTP and 100.9 kDa for the PfPKG-HA fusion protein. As a protein loading control, Pfαtubulin was detected using a mouse monoclonal antibody (Tat1). (D) Western blot of synchronised parasite cultures of the clone PfPKG-PTP-1A 24 hours (mostly mid trophozoites), 30 hours (mostly late trophozoites), 41 hours (mostly early schizonts) and 46 hours (mostly late schizonts) post invasion was detected with anti-ProtC and re-probed with an antibody against Pfαtubulin to estimate the relative total protein loading between lanes. (TIFF) [file pone.0048206.s002.tiff]

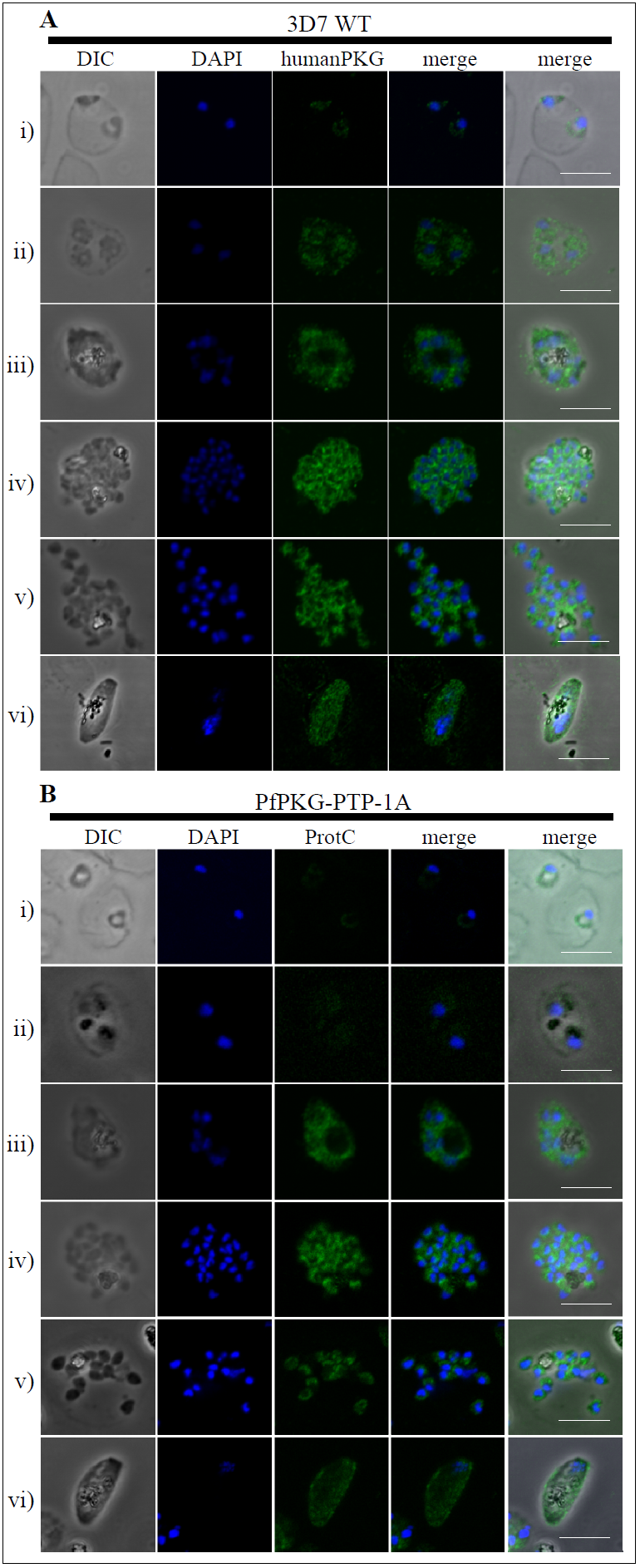

Supplement: Figure S3 — IFA of PfPKG in PfPKG-PTP-1A and WT 3D7 lines. Immunofluorescent detection using antibodies against (A) human PKG on WT parasites of the 3D7 clone and (B) ProtC on parasites of the PfPKG-PTP-1A clone. Representative images of (i) a ring stage parasite, (ii) two early trophozoites, (iii) an early schizont, (iv, v) late schizonts and (vi) a stage III gametocyte are shown together with bright field images (first column) and parasite nuclei stained with DAPI (second column). Bars ∼5 µM. (TIF) [file pone.0048206.s003.tif]

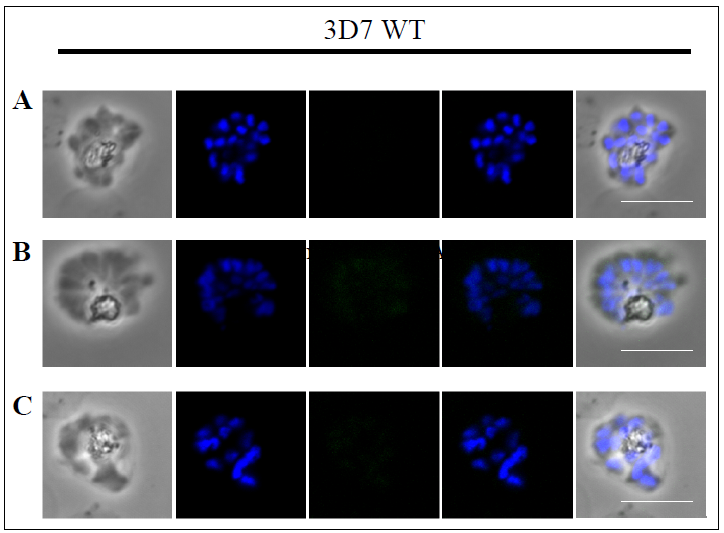

Supplement: Figure S4 — Negative control IFA. Immunofluorescent staining of WT parasites (clone 3D7) with (A) the rat HA-antibody, (B) the mouse HA-antibody and (C) the rabbit ProtC-antibody. Representative IFA images are shown together with bright field images (first column) and parasite nuclei stained with DAPI (second column). Bars ∼5 µM. (TIF) [file pone.0048206.s004.tif]

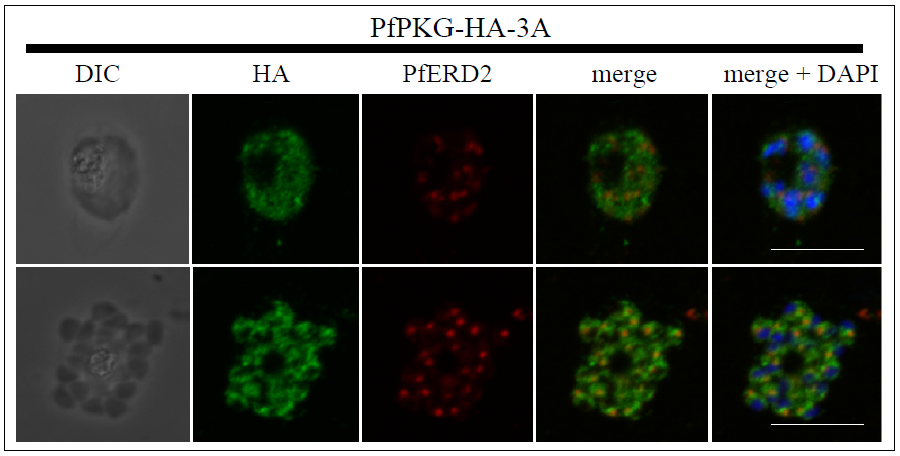

Supplement: Figure S5 — Dual staining of PfPKG-HA together with PfERD2. Dual staining of PfPKG-HA in fixed smears of early and late schizonts of the PfPKG-HA-3A clone together with PfErd2 [35]. Representative images are shown, together with bright field images (first column) and parasite nuclei stained with DAPI (in the merged image). Bars ∼5 µM. (TIF) [file pone.0048206.s005.tif]

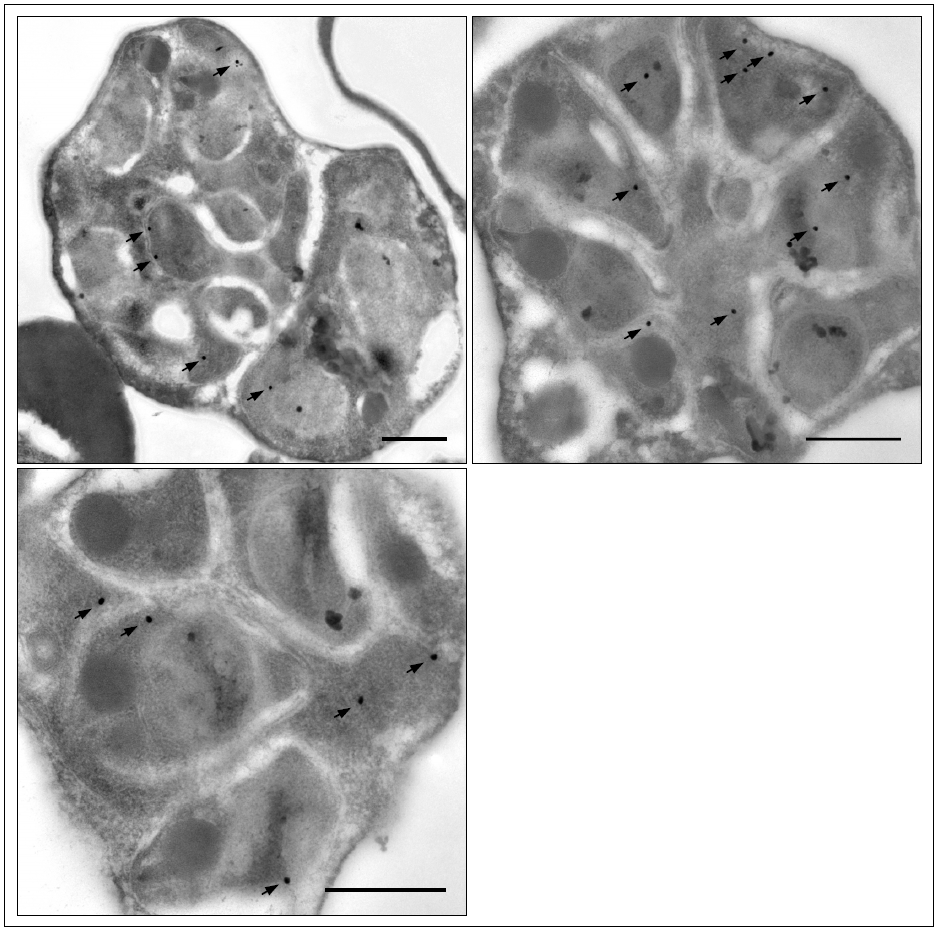

Supplement: Figure S6 — Immunoelectron microscopic visualisation of PfPKG-HA-3A. Segmented schizonts of the PfPKG-HA-3A clone were fixed in formaldehyde/glutaraldehyde and mounted in LR white resin. PfPKG-HA was detected with mouse HA antibody; secondary detection was performed with 10 nM gold particle-coupled anti-mouse antibody. Sections were counter-stained with uranyl acetate and images were captured digitally on a Jeol JEM –1200EX II electron microscope. Bars 500 nm. (TIF) [file pone.0048206.s006.tif]
