# Supplementary material for: Spatiotemporal and Functional Characterisation of the Plasmodium falciparum cGMP-Dependent Protein Kinase
Source: PLoS One. 2012 Nov 5;7(11):e48206. doi: 10.1371/journal.pone.0048206 (PMC3489689; doi:10.1371/journal.pone.0048206)
Supplement: Methods S1 — Additional details of experimental procedures. (DOCX) [file pone.0048206.s007.docx]

**SUPPORTING INFORMATION**

**Supplemental experimental procedures**

**Plasmid construction**

Transfection constructs based on the pHH1 vector [[22](#_ENREF_22)] were generated in order to C-terminally tag the endogenous *PfPKG* locus individually with a protein C-tobacco etch virus-protein A (PTP)- and haemagglutinin (HA)-tag. The constructs contained a 1.7 kbp C-terminal fragment of the *PfPKG* gene (to enable single crossover recombination) fused to the C-terminal tag and flanked by a 0.65 kbp fragment of the 3’UTR of *PfPKG*, and a human dihydrofolate reductase (*hDHFR*) gene, which confers resistance to the antifolate WR99210 for positive selection. The full length *PfPKG*, without its stop codon, had previously been amplified from *P. falciparum* cDNA using the primers 5’-CGCCCTAGGAAAA TCTATGTCCCAGTTGTCTTCATCG-3’ (*Avr*II site underlined) and 5‘-GCGG GATCCAAATGGAAGAAGATGATAATCTAAAAAAAG-3’ (*Bam*HI site underlined) and had been cloned into the pHH2 plasmid. Also, a 1.7 kbp C-terminal fragment of *PfPKG*, with its stop codon, had previously been amplified using 5’-CGCCCTAGGA AAATCTATGTCCCAGTTGTCT TCATCG-3’ (*Avr*II site underlined) and 5’-CGCA GATCTATGACTGAAGCT TTACAACAGAGTG-3’ (*Bgl*II site underlined) and had been cloned into pHH1. Using *Avr*II and *Bcl*I (which cuts within the *PfPKG* gene), the C-terminal 772 bp of *PfPKG* were excised out of pHH2 and cloned into the pHH1 plasmid containing the 1.7 kbp C-terminal fragment of *PfPKG*, thereby creating a 1.7 kbp C-terminal *PfPKG* fragment without its stop codon. A 0.65 kbp fragment of the PfPKG 3’-UTR was amplified in a nested PCR first using the primers 5’-GTAG CGGCCGCCCAACACCATTCAGAGGTTTATTAT-3’ (*Not*I site underlined) and 5’-TTGACATATGGGCATTGGGAATATGCC-3’, resulting in a 1 kbp-PCR product, which was used as a template for the second amplification step using the primers 5’-GTAGCGGCCGCCCAACACCATTCAGAGGTTTATTAT-3’ (*Not*I site underlined) and 5’-CGCCTCGAGATAAATAAATAAATAAA ATATATATATATATGTAGAG-3’ (*Xho*I site underlined), thus producing the final 0.65 kbp product, which was cloned downstream of the C-terminal PfPKG fragment into pHH1. The triple HA-tag was amplified from pHH1-HA3 [[23](#_ENREF_23)] using the primers 5’-GATCTCGAGTCAAGCGTAATCTGGAACGTC-3’(*Xho*I site underlined), and 5’-CATCCTAGGTACCCTTACGATGTTCCTGAC-3’ (*Avr*II site underlined). The PTP-tag was amplified from a plasmid obtained from the Arthur Günzl, University of Connecticut Health Center [[24](#_ENREF_24)] in order to introduce new cloning sites using the primers 5’-GCGCTCGAGTCAGGTTGACTTCCCCGCGGAA-3’ (*Xho*I site underlined), and 5’-GCGCCTAGGGAAGATCAGGTGGATCTTCGTC-3’ (*Avr*II site underlined). The triple HA and the PTP epitope tags, each ending with a stop codon, were cloned directly downstream in frame with the C-terminal PfPKG fragment, upstream of the 3’UTR. Prior to introducing the constructs into a *P. falciparum* culture, constructs were analysed using restriction digests to verify presence of inserts and resistance cassettes. Sequencing of the *PfPKG* fragment, the *PfPKG* 3’UTR and the PTP-/HA-tags was also carried out. A schematic of the tagging constructs is shown (Fig. S1).

**PCR and Southern blot analysis of transfected parasites**

After three cycles on/off WR99210 treatment, diagnostic PCR was performed to detect the presence of parasites in which successful single crossover has taken place. Genomic DNA was extracted from transfected parasites as described previously [[23](#_ENREF_23)] and was used in a PCR with primer sets that only amplify a product upon successful integration of the transfection construct into the *PfPKG* locus (Fig. S1). A primer specific for the HA-tag (5’-GATCTCGAGTCAAGCGTAATCTGGAACGTC-3’) and PTP-tag (5’-GCGCTCG AGTCAGGTTGACTTCCCCGCGGAA-3’) respectively, were used together with a primer binding to *PfPKG* upstream of the 1.7 kbp C-terminal *PfPKG*-region, that was part of the transfection construct (5’-GAACCAACCGCTTGTGCATCC-3’). PCR products were sequenced to confirm correct tagging of the *PfPKG* locus.

For Southern blot analysis of parasite clones, genomic DNA was digested with *Eco*RI and *Xba*I restriction endonucleases respectively, for probing against *PfPKG* and *hDHFR*. The *hDHFR*-probe was generated in a digest of a pHH1 transfection plasmid using *Bam*HI and *Hind*III restriction enzymes. The 795 bp-*PfPKG*-probe was amplified in a PCR using the pHH1-PKG-HA transfection plasmid as a template with *PfPKG*-specific primers (5’-GCGCTCGAGAAAATCTATGTCCCAGTTGTCTTCAT-3’; 5’-GAAAGAGAAATAA CAGCAGAAAATGATCATCC-3’).

**Immunoelectron microscopy**

Schizont stage cultures of *P. falciparum* 3D7 WT and PfPKG-HA-3A parasites were harvested by MACS and returned to culture for 8 hours until most schizonts were fully matured and segmented. After initial fixation in 4 % formaldehyde in RPMI 1640 (Invitrogen), schizonts were first transferred to 4 % formaldehyde in 1 x PBS and then to 4 % formaldeyde/0.1 % glutaraldehyde in 0.2 M HEPES. Samples were incubated in 50 mM ammonium chloride in 0.2 M HEPES, washed in 0.2 M HEPES and water before sequential dehydration in 30 % ethanol to 95 % ethanol. Samples were incubated in 95 % ethanol and 66 % LR white resin, transferred to 100 % LR white resin and then to gelatine capsules for anaerobic polymerisation. Ultrathin sections (100-120 nm) were cut on a Leica Ultracut R microtome and mounted on pioloform-coated grids and used for immunoEM. Primary mouse HA antibody (clone 16B12, Convance) was diluted 1:250 in PBFNT buffer. Secondary detection was performed with 10 nM gold particle-conjugated goat anti-mouse IgG (BBI Life Sciences). Sections were counter-stained with 2 % (w/v) aqueous uranyl acetate. Images were captured digitally on a Jeol JEM – 1200EX II electron microscope.

**Antibodies**

In IFA, rabbit anti-ProtC (ab18591-200, Abcam) was used at 1:50 and polyclonal rabbit anti-PfERrd2 (obtained from the Malaria Research and Reference Reagent Resource Center, MR4) was used at 1:1000.
